# Evaluation of small hydropower turbines installed downstream of Nile River branches (Egypt)

**DOI:** 10.1038/s41598-023-41775-1

**Published:** 2023-09-12

**Authors:** Mohamed E.A.E. Ahmed, M. Attia Abdellatif, Ahmed A. A. Attia, Ahmed Farouk Deifalla, Mostafa E. A. Elsayed, M. A. Abdelrahman

**Affiliations:** 1 Combustion and Energy Technology Lab., Mechanical Engineering Department, Shoubra Faculty of Engineering, Benha University, Cairo, 11672 Egypt; 2 National Water Research Center, Mechanical and Electrical Research Institute, Ministry of Water Resources and Irrigation, Delta Barrages, Cairo, Egypt; 3 Structural Engineering and Construction Management, Future University in Egypt, Cairo, Egypt

**Keywords:** Mechanical engineering, Power stations, Environmental sciences

## Abstract

The current study proposes a new strategy for using small hydroelectric turbines in downstream river branches with the least amount of construction and the lowest cost by comparing two different methods of installing the turbines, the first by installing the turbines at the river's bottom and the second by installing the turbines on floating boats. The methodology of this article is based on predicting the distribution of velocities through the watercourse using experimental data collected at various points in the river's depth, and then predicting the resulting electrical power for different sizes of turbines, as well as estimating the number of turbines for each row and the number of rows along the river. Therefore, Investigate the proposed systems. The proposed small hydropower system's economic viability and environmental impact are investigated in this article. According to the nature of the waterway, the best diameter of a turbine that can be used is 1.5 m based on water velocities and river depths. The proposed power plant generated 25.8 kW per single turbine row, with an estimated cost of produced power (0.035 USD/kWh) of approximately 20 turbines installed per row. Compared to other renewable energy sources, the proposed hydropower system is cost-effective and environmentally friendly, as generating electricity with the proposed small hydropower plant could reduce annual carbon dioxide emissions by 368 tones of CO_2_ per single turbine row.

## Introduction

Most commercial and industrial operations necessitate the use of electrical energy. Energy generation is vital to the economic and social growth of both developed and developing countries. Rapid economic growth requires increased energy generation, but the challenge is to sustain the desired energy. Hydro-energy technologies provide fascinating non-polluting alternatives to the existing reliance on fossil and nuclear-fueled power plants to meet the increasing demand for electrical energy. The seas and rivers constitute a massive energy reservoir of stored solar and gravitational energy that may be used in a variety of ways. This energy is normally dissipated but, in many cases, far more intense than other kinds of renewable energy that are currently effectively utilised on land. Wave energy, water current energy, tidal barrages, and osmotic pressure variations are all potential sources of offshore energy. Both wave energy and marine-current energy convert the kinetic energy of flowing water to electricity without the use or diversion of traditional hydroelectric facilities based on dams or penstocks and hence fall under the category of hydrokinetic energy conversion. Tidal barrages, like conventional hydroelectric dams on land, utilise potential energy from height disparities. Hydrokinetic techniques are intended for use in streams that exist naturally such as rivers, tidal coastlines, and ocean currents, as well as in some artificial waterways (for example, canals). Small hydropower can contribute to global energy, mainly in developing nations. Micro-hydropower has the potential to provide more energy than solar photovoltaics. Africa has one of the world's lowest hydropower utilization rates. While hydropower generation development is becoming more challenging due to environmental and socioeconomic concerns and the resource's vulnerability to changing climates and water supply in major waterways, micro-hydropower development remains a viable option. Mini hydro (sometimes defined as having an installed capacity of less than 1 MW) and micro-hydro (below 100 kW) are two types of hydropower^1^. According to the Egyptian Ministry of Electricity and Energy (2021), Egypt's hydropower energy capacity reached 2832 megawatts out of a total energy capacity of 60,000 MW^2^. Many research papers have been presented to increase the energy extracted downstream (away) from power plants from dams using various principles and criteria of the applied approach, the expected exploited energy, and the assembly of small plants to optimize the utilization of the riverbed.

Shafei et al.^3^ laid the groundwork for a farm of hydrokinetic turbines on the stilling basin of the barrage's gate spillways, where the barrier collected 14.88 MW of total electric production, compared to the conventional hydropower plant's rated value of 32 MW. Lalander and Leijon^4^ studied the effects of using Stream Energy converters in rivers using an analytical and numerical model. The analytical model described the increase in water level caused by energy capture as a function of how much the turbine has blocked the channel. When the turbines cause drag on the flow and energy is lost in wake mixing, the significant head loss has also been demonstrated to be the difference between energy capture and energy losses. India's water resources and minor hydropower potential have been evaluated. By Himanshu Nautiyal et al.^5^ India's total small hydro potential is 15,000 MW, but only 16% of this potential has been developed for power generation. Adejumobi and Shobayo^6^ devised a method for determining the best small hydropower turbines for optimum power output that can be used yearly. The system had a mean head of 37 m and a retained flow of 2.97 m^3^/s. According to the study's findings, the only way to optimize the energy output from any chosen Small Hydro Power site is to have a thorough technical knowledge of turbine selection. Tomporowski et al.^7^ investigated the pressure, force, and torque characteristics of floating hydro turbines numerically, and the calculated forces and moments show that the depth of the channel has a significant impact on the results of the moment of force and torque growth. According to Winter^8^, despite their much smaller size, tidal turbines produce significantly more oversized loads than wind turbines of similar power (at least during regular operation). This explains why tidal turbine blades are mainly built of carbon composite materials. However, wind turbine blades are mostly made of glass fibre. Even though wind speed fluctuation is far more significant than tidal flow speed fluctuation, it has been established that angular velocity changes for a tidal turbine are more significant than for a wind turbine. Hiromichi Akimoto et al.^9^ developed a new water flow turbine concept called turbine support. The direction of the turbine axis is not fixed in this concept, and the tilt angle is passively adjustable to the stream velocity. They showed that the concept's energy cost is comparable to that of a land-based wind turbine. Ramadan et al.^10^ created and tested an S-shaped water current stream energy conversion system. The numerical simulation revealed that for a tip speed ratio of 0.8 and a streamflow velocity of 3 m/s, the S shape blade has a maximum power coefficient of 24.6%, representing a 40% improvement. In contrast to the conventional design. Wen-Quan et al.^11^ investigated the performance of a micro-hydrokinetic river turbine with a horizontal axis. According to the findings, the rotor performance does not degrade even when the current speed varies, or the TSR deviates from the design values. Furthermore, they discovered that the greater the pitch angle, the smaller the axial force coefficient, implying that when the flow velocity is too high, the pitch angle is increased to reduce the axial load, ensuring the river turbine's overall stability. Kumar and Saini^12^ used both an experimental and numerical approach to investigate the performance of a Savonius water turbine in an open channel. According to the numerical analysis, the turbine had a maximum power coefficient (C_P_) of 0.23 at a tip speed ratio of 0.70, whereas an experimental investigation revealed a maximum C_P_ of 0.21 at a tip speed ratio of 0.72. Earlier research has shown that placing various types of turbines in streams and rivers can provide a reasonable quantity of energy. The studies also discussed the feasibility of installing various types of turbines in supply networks due to flow and pressure caused by fluctuating demand throughout the year, affecting turbine vehicles' energy efficiency. None of the studies addressed the potential behind gated barrages, where high constant flow and constant high pressure are available, as will be discussed in this paper.

The following is a premier study to evaluate the techniques that use small hydropower turbines in Nile River Branches. This study is used to evaluate the availability of power output from the suggested system and discuss the different scenarios to determine the most suitable to be used with using the Nile River branches in goods transportation. Also, the study experimentally evaluates the reading of velocity profiles that were collected in one of the most famous branches of the Nile River (Menoufia Branch) just after the barrage.

## Methods

**Figure 1:**
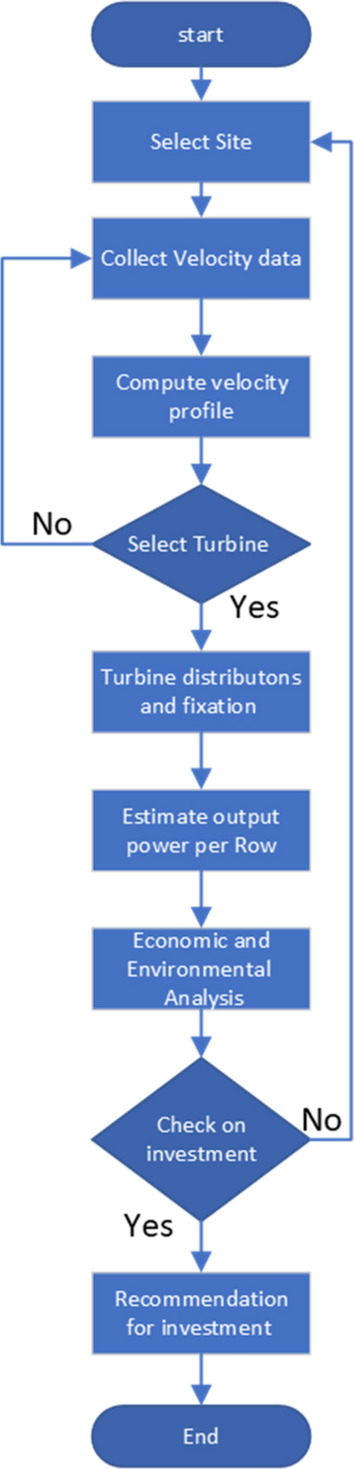
Technical diagram of the current study.

The current study proposes a new strategy for using small hydroelectric turbines for Delta Barrages Egypt (Menoufia Branch), where high flow rate and high-pressure values are available to produce up to 0.75 megawatts from a single station with the least amount of construction and the lowest cost. The current investigation was conducted in eight steps, as illustrated in the technical diagram in Fig. 1. Two scenarios were tested to install the turbine. The first scenario had the turbine installed at the river's bottom, while the second had the turbine installed at the base of a floating boat. Furthermore, all criteria were considered in this study to determine the economic feasibility of installing water turbines in the Nile Delta barrages for energy production, as well as the environmental impact of the current proposal.

### Calculation procedure

**Figure 2:**
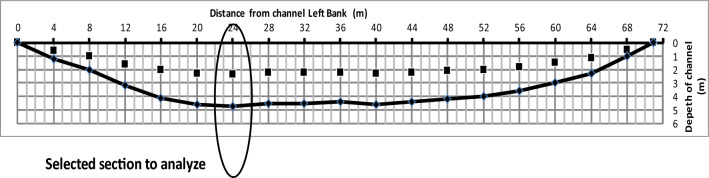
The distribution of the measured points along the channel width starts from the left bank.

The data provided for the selected section of the Nile River (Menoufia Branch) is a measurement of the flow velocity in the middle of the depth of the channels. The depth of the measured point (h) is changed according to channel depths (H) at the selected section. The velocity measurement process is briefly explained in the next section. Measured velocity points were drawn to obtain the shape of the velocity profile across the selected section of the channel as shown in Fig. 2. These measured points have been analyzed using a system of equations explained in the next section of the paper to obtain velocity profile across the channel and easily determine how much power can be produced using our proposed system.

**Figure 3:**
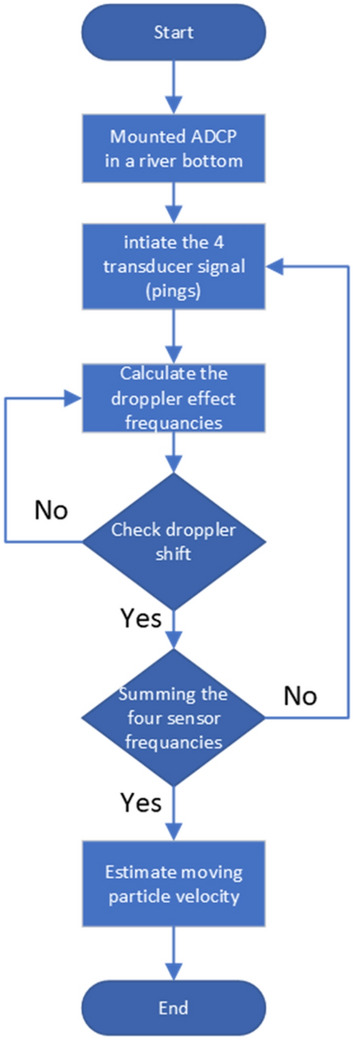
ADCP water velocity measurements flow chart.

**Figure 4:**
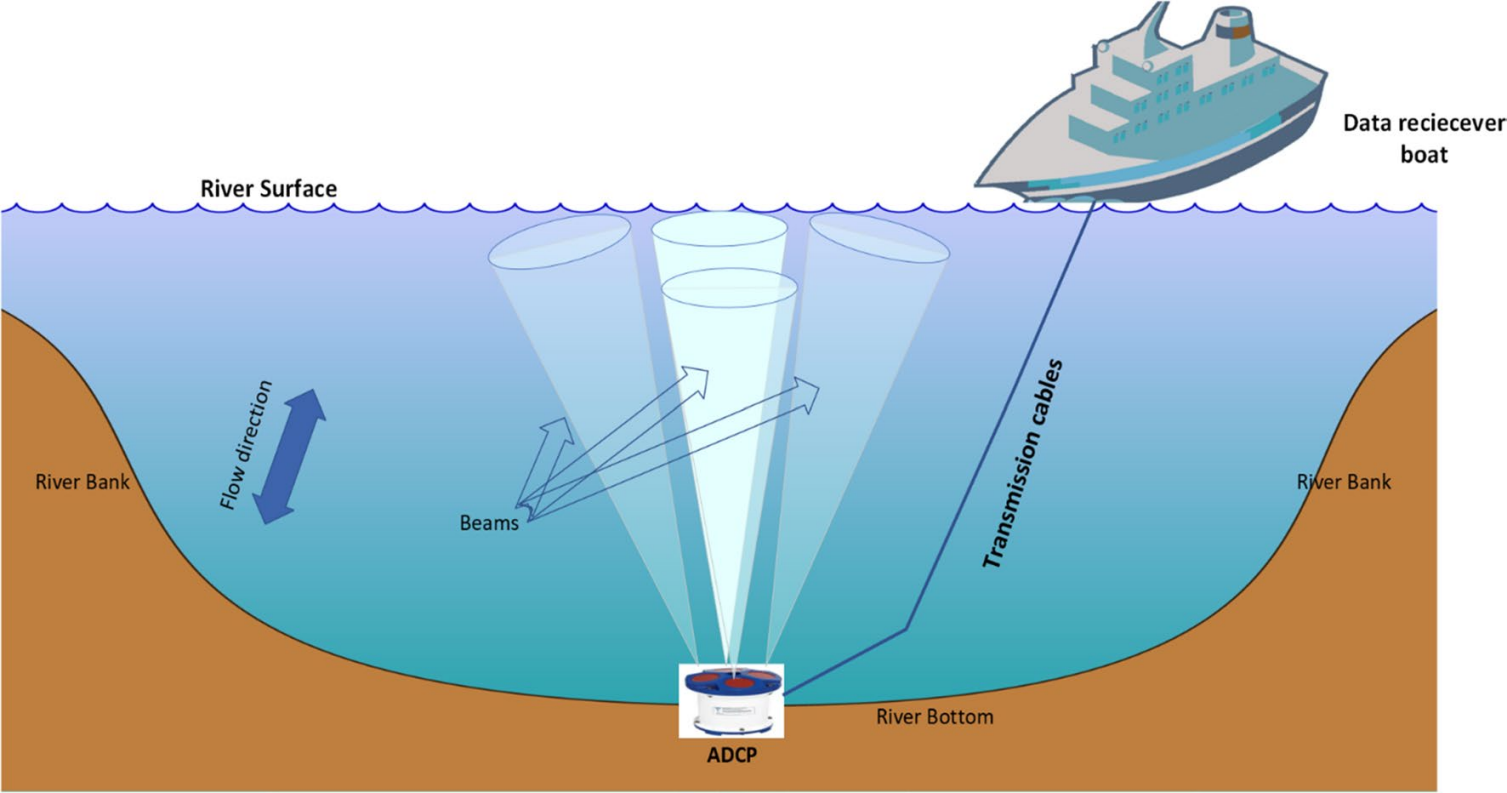
System description for water velocity measurements.

An Acoustic Doppler Current Profiler (ADCP) detects water currents by utilizing the Doppler effect and properties of sound waves. The ADCP works by emitting "pings" of sound into the water at a predetermined frequency, as they pass, the sound waves bounce off the suspended particles in the rushing water and return to the instrument^13,14^. Because of the Doppler effect, the frequency of sound waves reflected from a particle moving away from the profiler is slightly reduced. The Doppler shift is the frequency difference between the waves emitted by the profiler and the waves received by it. The device uses this shift to determine how fast the particle and the water surrounding it are moving by using the flow chart, as shown in Fig. 3. Bottom-mounted ADCP, batteries and an internal data recorder require an anchor to keep them in the required place, as shown in Fig. 4. A vessel with power and an onboard computer to receive the data are requirements for vessel-mounted equipment^15^.

**Figure 5:**
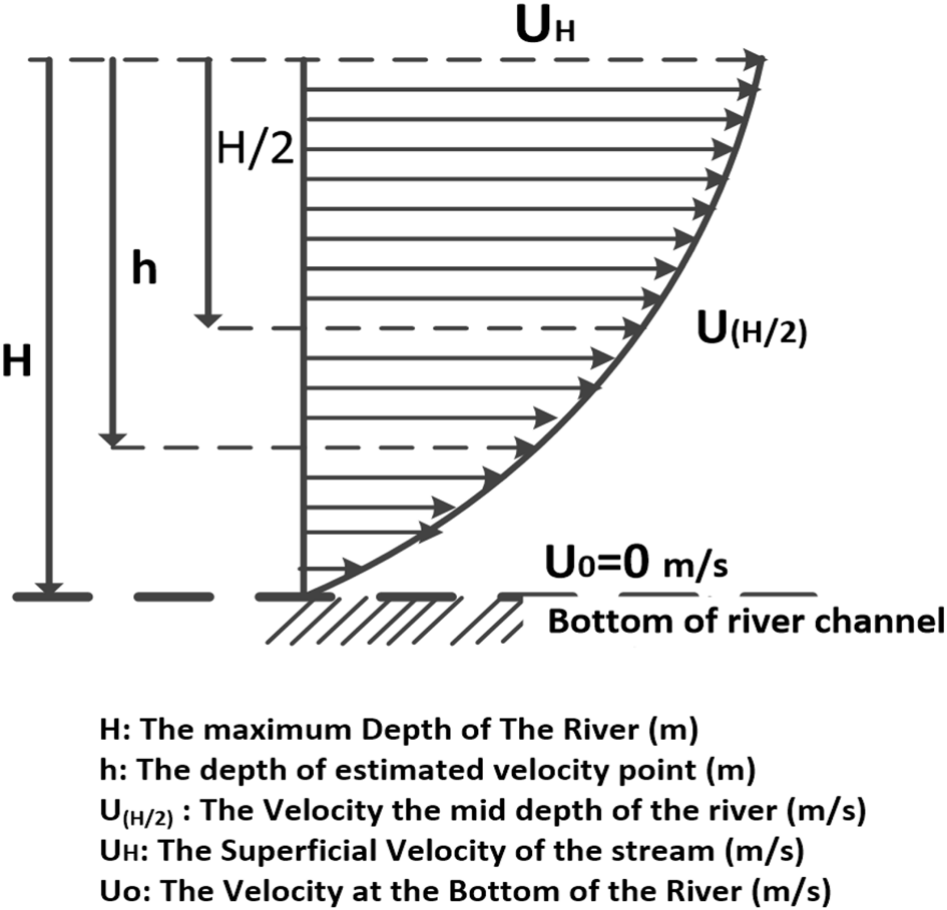
Velocity profile variation with depth.

Field measurements were analyzed using a 2D mathematical model to obtain the velocity profile and average velocity at the channel's half depth, and the model was then solved using Microsoft Excel software. the average velocity through the section at every 4 m as steps from the left bank of the channel can be obtained by calculating the superficial velocity using the above equations and with the measured value of the velocity of the section in the half depth of the illustrated location as shown in Fig. 5. The superficial velocity (U_H_) needs to be estimated to calculate the average flow speed in the following procedure of estimating the superficial velocity from the measured velocity at half of the depth and the channel depth at each location. The velocity distribution along the depth could be expressed in Eqs. (1)–(3). The average velocity of the upper part of the stream from the middle of the depth to the superficial surface can be calculated using Eq. (4). The output power is primarily determined by the flow velocity of the upper part of the stream using Eq. (5).

1 $$U={C}_{1 }\mathrm{ln}\left(\frac{h}{H}\right)+{C}_{2}$$

At h = H U = 0 so that the constant C_2_ = zero

$$At \, Depth= \frac{H}{2} \quad \quad Flow \, Velocity = {U}_\frac{H}{2}$$

2 $$\mathrm{Then } \quad \quad {C}_{1}=\frac{{U}_{H/2}}{Ln\left(0.5\right)}$$

3 $$U= \frac{{U}_{H/2}}{Ln\left(0.5\right)}\mathrm{ln}\left(\frac{h}{H}\right)$$

4 $${U}_{ave}= \frac{{U}_\frac{H}{2}+{U}_{H}}{2}$$

5 $$P={0.5C}_{p}\rho A{U}_{ave}^{3}$$

**Figure 6:**
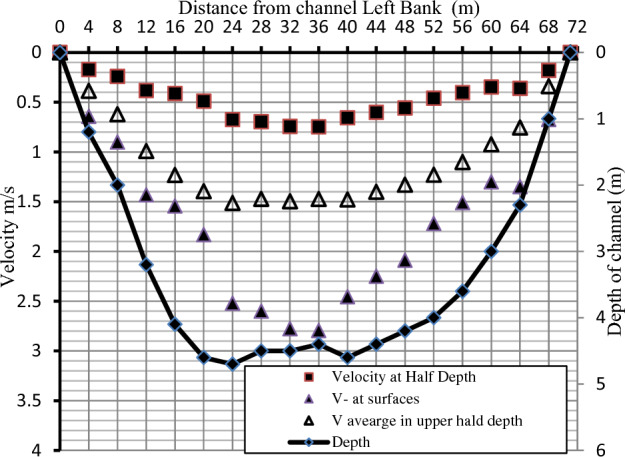
The estimated velocity profile and depth of the selected section.

According to Betz^16^, no more than 59.3% of the available water energy can be converted into mechanical energy under ideal conditions. Modern turbines can convert energy at a power coefficient Cp ranging from 0.4 to 0.5 under actual operating conditions and with the advancement of aerodynamic technology^17^. The current study was based on high-efficiency turbines with a C_p_ of 0.5. Figure 6 shows that the waterway's depth ranges from 0 to 4.7 m as the velocities increase from the bottom to the surface. It is also clear that they are limited in the middle of the depth, ranging from 0 to 0.7 m/s. Because of the low water velocity in the lower half of the stream's depth and the presence of shallow water, rocks, sediments, and swirls caused by the wall boundary layer, all these factors preclude the installation of a water turbine in this area.

On the contrary, large water velocities ranging between 0 and 2.7 m/s can be observed in the upper part of the depth, in addition to the fact that the stream of water is uniform and free of eddies and obstacles, all of which makes it logical to use the upper part of the depth of the stream to generate electricity via water turbines. Given that the water depth in the upper half of the stream ranges from 0 to approximately 2.35 m, installing turbines across the entire width of the waterway is not feasible. A 20-m buffer should be left on both sides of the stream where the water depth increases from 2 to 2.35 m.

**Figure 7:**
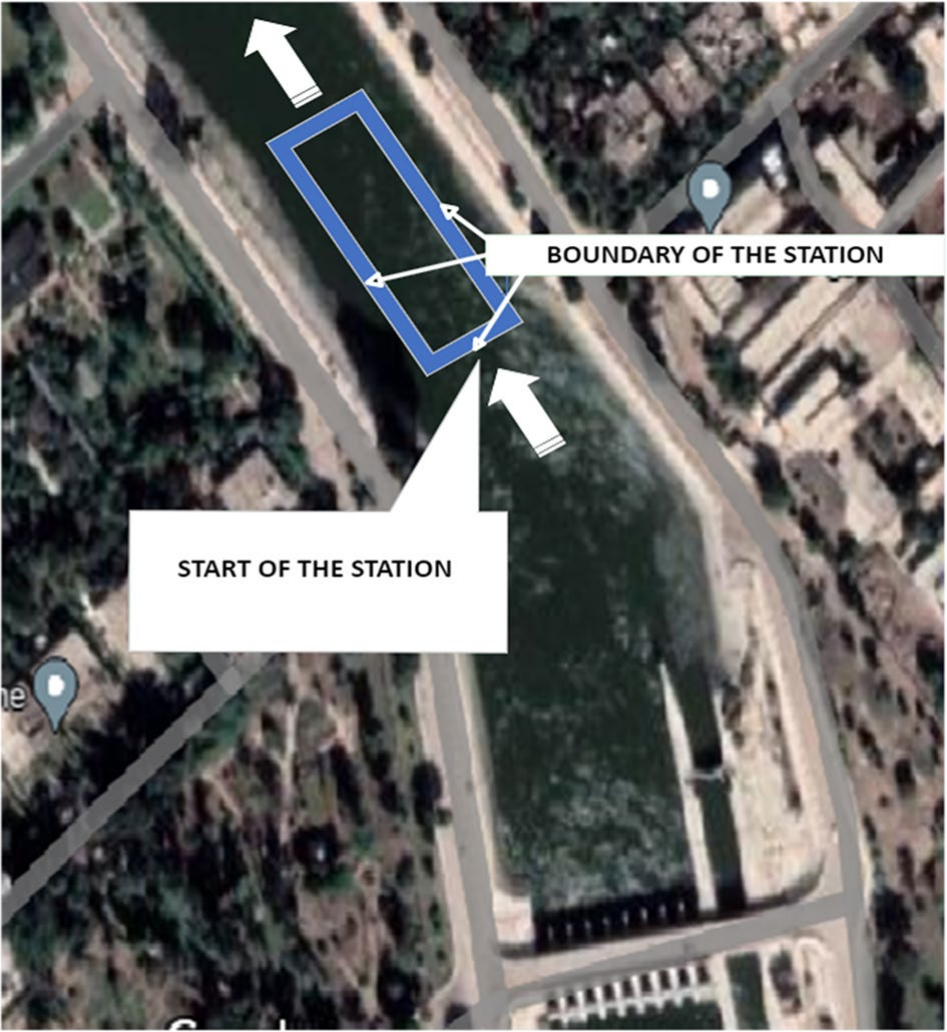
Map of the selected Nile River branch with the location of the proposed station. (Taken from Google Earth, https://earth.google.com/web/search/Nile+river) location of the station placed on the map by the authors.

A 20-m buffer zone on both sides of the waterway can be used for river navigation without interfering with the turbines installed in the stream, as shown in Fig. 7. As a result, the stream's net width is approximately 32 m in the middle, with water depths ranging between 2 and 2.35 m in the upper half. This is the proposed distance from the width of the watercourse to install the water turbines, where it was proposed to install several sizes of water turbines ranging in diameter from 1 to 2.5 m to achieve the optimum turbine diameter for this type of waterway.

**Figure 8:**
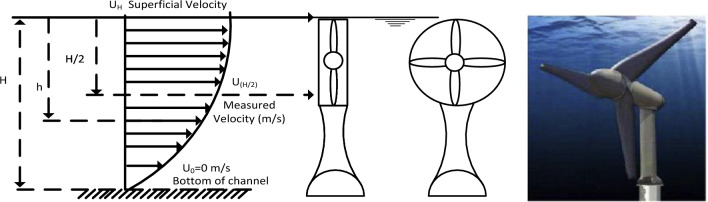
Base supports for the turbine station. Verdant Power's Kinetic Hydro Power System^18^.

**Figure 9:**
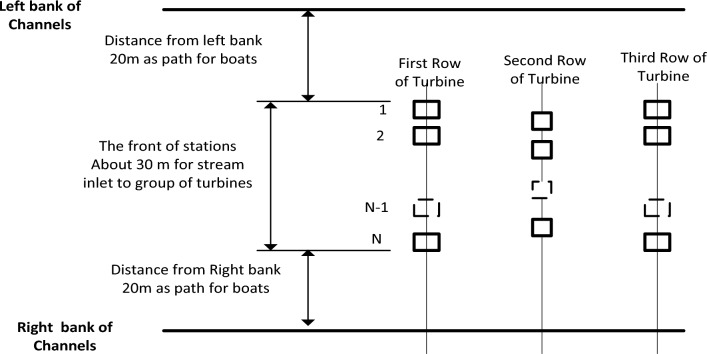
Distribution of turbine groups on the station area through the channel to increase the usability of the energy when using the base supports technique.

Configurations of turbine stations in the channel have two scenarios, First Scenario (Base Supports for the turbine). For this scenario, tidal turbines are chosen and installed at the channel's bottom, with the rotor oriented to face the flow at a higher velocity and extract energy from the flow, as shown in Fig. 8. The height of the water heavily influences this turbine configuration in the channel; when the height of the water drops to the point where the entire rotor is only partially submerged, the turbine cannot extract energy from the flow. To install this configuration efficiently, it must first indicate the location of higher flow velocity because it will be mounted at the bottom and changing its location will incur a high cost. There is another constraint for navigating boats across the river. The second scenario will alleviate these constraints. Figure 9 depicts a line diagram for the distribution of turbines in a section of the Nile River. A 20-m path was also left on each bank of the channel to allow boats to cross the turbine section and reach the turbine station easily. After leaving these paths, there is a 30 m length in the centre of the channel ready for installing turbines, as the width of the selected channel is about 70 m.

**Figure 10:**
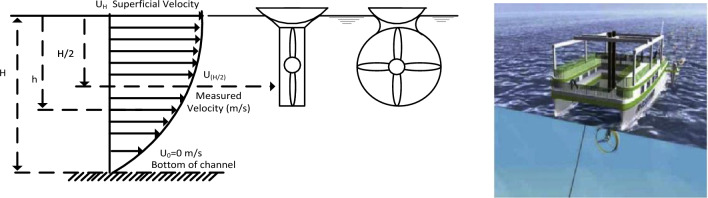
Boat supports for the turbines. The FRI-EL Sea Power Concept: floating platform (taken from Burgermeister, 2008)^19^.

In the Second Scenario Boat was supported for turbines. In this scenario, turbines are attached to a floating device or a boat, and the rotor is directly submerged in water, as shown in Fig. 10, consuming flow energy as previously mentioned, the higher flow velocity obtained close to the half-height of the river. This fixation method is simple to maintain, low in cost, does not require complex construction, and is not affected by water height. This fixation method allows for greater flexibility in changing the location of turbines across the river to obtain maximum energy without incurring additional costs or requiring reconstruction. Recommended distribution of turbines into three rows, each row containing (20) turbines capable of producing up to 0.075 MW of electrical energy; using the ten stations will be about 0.75 MW to avoid blocking the river as three rows require 30m distance along the river for a turbine to be placed (as it required to leave between rows a distance of 10 D (D: diameter of the installed turbine) to avoid wakes after first row affected second row). This scenario can be repeated indefinitely along the river for maximum utilization of flow energy without any harmful effects on the environmental situation, as there is no construction (Fixation block of turbine for first scenario on the bottom of the river) on the bottom of channel which affected marine life, block the base flow, as the area available for following base flow is decreased. This scenario allows for increasing the number of turbines per row across the river, without affecting the life of fish or other animals that need to swim across the turbines.

When compared Both scenarios provide the same amount of power, however, the first is not recommended for this location due to its negative environmental effects, high initial cost, and numerous, well-explained constraints in the current work. The second scenario is anticipated to be more reliable because of its low cost, simple installation, and environmental friendliness. If the flow velocity decreases, we can also quickly alter the station's location by transferring the boats to a new area, unlike the first scenario, which is not conceivable.

## Results

**Figure 11:**
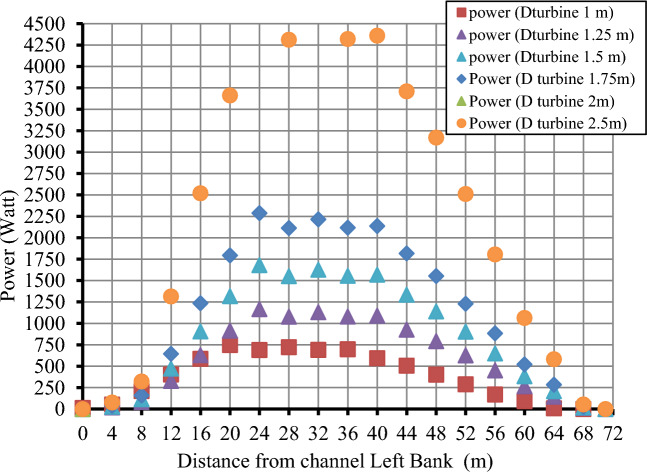
The turbine's estimated power (watt) at various locations along the channel width.

**Figure 12:**
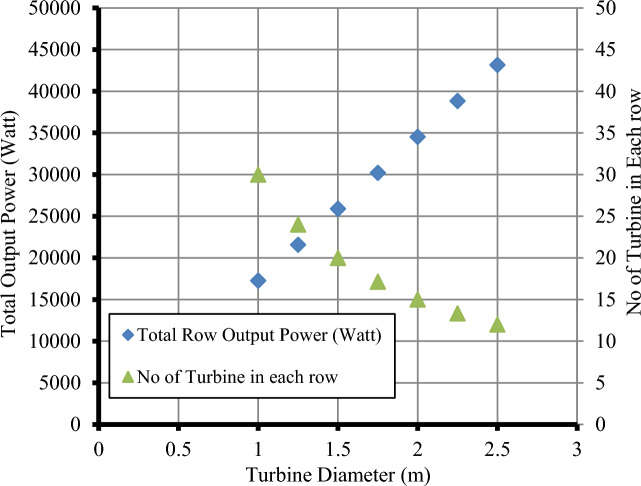
Total output power produced by a single row of turbines.

Both scenarios described above can extract flow power, but each has limitations based on operating channel criteria (maximum and minimum water depths in the channel). The estimated power from the turbine with different diameters at the previous location of velocity measurements is shown in Fig. 11 and calculated for turbines with different diameters based on the water flow velocity using Eq. (5). According to the positions of each turbine intended from the left, it could be concluded that the statistically significant output power will be about 20 m for the lift bank of the canal and end at about 20 from the right bank. That means the mainstream users will be in the mid-section of the canal, starting from 20 to 50 m from the left bank of the canal. With the increase of turbine diameter, the output power increased. However, due to the canal's depth, shown in Fig. 12, there is a limitation on turbine sizes that will be allowed to be used.

The total power produced by a row of turbines with different diameters is shown in Fig. 12. Based on the shape of the channel, 20 turbines with a diameter of 1.5 m are selected to extract the maximum available flow power, but the selected turbine does not produce maximum power. However, it is the most suitable turbine for the channel section to satisfy the maximum and minimum water depth. By dividing the net distance from the width of the waterway by the different turbine sizes, the maximum number of turbines for each size that can be installed in the water stream can be obtained, as shown in Fig. 12, where 30 turbines with a diameter of 1 m can be installed, while 12 turbines with a diameter of 2.5 m can be installed. The water's average velocity in the stream's upper part can be calculated using Eq. (3), and the turbine's output power can be calculated by substituting it in Eq. (4), assuming that the performance coefficient for all turbine sizes is 0.5. As the turbine output power is directly proportional to the square of the turbine diameter, increasing the diameter of the proposed turbine from 1 to 2 m increases the total expected output power from 17.26 to 43.16 kW. The curve shows that using 12 turbines with a diameter of 2.5 m produces more power than using 30 turbines with a diameter of 1 m, and thus it can be predicted that the best turbine diameter for the waterway is 2.5 m. However, it requires at least 50 cm of depth for installation after considering how to install the turbine. Because the net depth at which the turbine can be installed varies between 2 and 2.35 m, deducting the necessary distance for installing the turbine from the available depth yields a net depth of 1.5 to 1.85 m. As a result, if a turbine with a diameter of 2.5 m is placed, large portions of the blades will fall in areas of reduced water velocity, causing the turbine's performance to deteriorate and unable to work with a performance coefficient of 0.5. As a result, we can conclude that the 1.5-m-diameter turbine is the best option in these conditions for achieving total performance efficiency. As a result, a single row of 20 turbines with a one-meter diameter can generate 25.8 kW of electricity.

## Discussion

### Economic analysis

The key parameters used in the present research for economic planning were Net Present Value (NPV) and Levelized Cost of Electricity (LCOE). The project is feasible if the NPV is more significant than zero calculated based on Eq. (6). The LCOE value is linked to the minimum market sales rate for the energy required to make the project feasible^20,21^.

6 $$NPV= \sum_{t=1}^{m}\frac{E.T-{C}_{OM}}{{\left(1+i\right)}^{t}}-I$$

7 $$LCOE=\frac{{\sum }_{t=1}^{m}\frac{{C}_{n}}{{\left(1+i\right)}^{t}}}{{\sum }_{t=1}^{m}\frac{E}{{\left(1+i\right)}^{t}}}$$

where: C_n_ = Annual Cost (USD); E = Energy produced annually (MWh/y); T = energy sales rate (Small hydropower = 64.27 USD/MWh) as shown in Table 1^22,23^, m = lifespan set to 20 years; i = Annual discount rate; t = year; C_OM_ = operating and maintenance cost in USD/year, and I = initial USD investment, calculated from the product between the unit cost (Cun) the installed power. Operation and maintenance cost = 1–4% of the initial investment and Discount rate = 9–11%. Total energy produced annually from 1 row = 177 MWh/y, initial investment based on installation and unit cost for 1 row around 104,000 USD. (Based on unit cost with installation from 4000 to 6500 USD/kW^24^. The maintenance cost will be taken as 4% of the initial investment as shown in Table 2, (for our case equal 4160 USD) and the discount rate as 10% (for our case equal 10,400 USD) by substitution in Eq. (7), LCOE for one row of hydraulic turbines has been calculated based on our proposed system with a value of 35.2 USD/MWh or 0.035 USD/kWh and this value falls within this range and appears reasonable, according to Table 1^,^ based on the plant's life span of 20 years^25^.

**Table 1:** Levelized cost of electricity (LCOE) estimate for energy resources entering service in 2027^23^.

Energy resource	Capacity factor (%)	Total LCOE, including tax credit
Conventional		
Ultra-supercritical coal	85	$82.61
Combined cycle	87	$39.94
Advanced nuclear	90	$81.71
Renewable		
Geothermal	90	$37.62
Biomass	83	$90.17
Wind, onshore	41	$40.23
Wind, offshore	44	$105.38
Solar, standalone	29	$33.83
Solar, hybrid	28	$49.03
Hydroelectric system	54	$64.27

**Table 2:** Typical installed costs and LCOE of hydropower projects^25^.

	Installed costs (USD/kW)	Operations and maintenance costs (%/year of installed costs)	Capacity factor (%)	Levelized cost of electricity (2010 USD/kWh)
Large hydro	1050–7650	2–2.5	25–90	0.021–0.19
Small hydro	1300–8000	1–4	20–95	0.021–0.25
Refurbishment/upgrade	500–1000	1–6		0.01–0.05

### Carbon mitigation and climate improvement

As most governments seek to adapt to and mitigate the adverse effects of climate change through the Conference of the Parties to the United Nations Framework Convention on Climate Change, sustainable development and environmental preservation have recently risen to the top of most countries' national agendas. Water turbines produce the same amount of energy as traditional polluting techniques, so the reduction in carbon emissions caused by using them to generate electricity can be calculated. According to the Environmental Protection Agency (EPA)^26^, the average carbon dioxide emissions for electricity generation methods based on fossil fuels ranged from 0.499 kg CO_2_/kWh for natural gas power plants to 1.012 kg CO_2_/kWh for coal power plants in 2020, as shown in Table 3.

**Table 3:** Summary of CO_2_ emission intensity.

Conventional electricity generation method	Low	Mean	High
	Kg CO_2_/kWh		
Coal	0.861	1.012	1.492
Petroleum	0.721	0.966	1.23
Natural gas	0.362	0.499	0.891

As a result, the CO_2_ reduction in tonnes per year per single row of water turbines is calculated using Eq. (8) ^27^:

8 $${\varnothing }_{C{O}_{2}}=\frac{{P}_{one Row}\times {\psi }_{C{O}_{2}}\times t\times 365}{1000}$$

where $${\psi }_{C{O}_{2}}$$ Is the average CO_2_ emission for coal-fired power generation (1.012 kgCO_2_/kWh),$${\varnothing }_{C{O}_{2}}$$ is the CO_2_ mitigation per year of a single row of water turbine (tCO_2_/annum.row), $${P}_{one \, Row}$$ Is the single-row output power (kW), and t is the operating hours, which are assumed to be 24 h per day. Assuming that the annual operating rate of the water turbines is approximately 78%^27^, the annual reduction in carbon emissions from one row of turbines can be estimated to be approximately 179 tonCO_2_. According to the National Bank^28^, carbon prices should be in the USD 50–100/tCO_2_ range by 2030 to encourage investors to switch to clean renewable energies and limit global warming to 2 °C. Thus, in the current carbon price analysis, the cost of carbon was taken as 50 $/tCO_2_.As a result, the cost of CO_2_ mitigation can be calculated from Eq. (9).

9 $${Z}_{{Co}_{2}}={C}_{{Co}_{2}}\times {\varnothing }_{C{O}_{2}}$$

where $${Z}_{{Co}_{2}}$$ Denotes the enviro-economic cost of CO_2_ mitigation per annum per row of selected water turbine ($/annum.row) and $${C}_{{Co}_{2}}$$ Denotes the carbon price per tCO_2_.

As a result, in addition to generating sustainable electrical energy from the watercourse and creating jobs. The proposed system is environmentally feasible, as one row of turbines can reduce approximately 179 tonnes of CO_2_ emissions that would have been produced if conventional fossil fuel energy had been used to generate the same amount of electricity. as well as saving $8950 per year that would have been spent on obtaining a comparable amount of electricity.

## Conclusion

The current study investigates the feasibility of using small hydropower turbines to generate electricity from the Nile Delta barrage (Monfia branch) in Egypt via two proposals for turbine installation, one fixed at the river's bottom and the other floating via a floating boat. The results of analyzing the distribution of water velocities and river depths concluded that the best diameter of a turbine that can be used according to the nature of the waterway is 1.5 m, with approximately 20 turbines installed per row and a production capacity of approximately 25.8 kW of electricity. Economically, the proposed small hydropower system has a Levelized cost of electricity of about US $0.035/kWh based on a plant life of 20 years, which is promising compared to other renewable energy resources. In addition to its role and importance in protecting the environment and generating electricity without green gas emissions, the proposed hydropower system has the potential to reduce carbon dioxide emissions by the equivalent of $8950 per row of water turbines each year, which is an additional incentive to encourage investment in the Renewable hydropower sector. Finally, this study served as a model for future in-depth research into the technology for incorporating water turbines into comparable systems. The study also emphasized the theoretical possibility of producing a permissible amount of electricity in the Nile Delta barrages and the financial and environmental implications.

## Data Availability

All data generated or analyzed during this study are included in this published article. All the material is owned by the authors and no permissions are required.

## List of symbols

A: The swept area of the water turbine rotor, m^2^
C_1_, C_2_: Constants
C_P_: Power coefficient of the turbine
C_n_: The annual cost, $
C_un_: Turbine unit cost, $
C_co2_: Carbon price, $/tCo_2_
E: The energy produced annually, MWh/year
H: Depth of water channel, m
H: Local depth of water channel at the given location, m
I: Initial USD investments, USD
i: Time, years
m: The life span of the water turbine, year
T: Energy sales rate, USD/MWh
U: Water flow velocity, m/s
U_ave_: The average water flow velocity, m/s
U_H_: Surface water flow velocity, m/s
$${\mathrm{U}}_{\frac{\mathrm{H}}{2}}$$: Flow velocity at a half depth of the water channel, m/s
$${\mathrm{Z}}_{{\mathrm{Co}}_{2}}$$: Enviro-economic cost of Co2 mitigation per annum, $/annum

## Greek symbols

$\rho$: Water density, kg/m^3^
${\psi }_{C{O}_{2}}$: Average Co_2_ emission for fossil fuel power generation, kgCO_2_/kWh
${\varnothing }_{C{O}_{2}}$: Co_2_ mitigation per year of a single row of water turbine, tCO_2_/annum.row

## Abbreviations

COM: Operating and maintenance cost, $/year
EPA: Environmental protection agency
KHPS: Keio household panel survey
LCOE: Levelized cost of electricity
NPV: Net present value
